# The DOA Estimation Method for Low-Altitude Targets under the Background of Impulse Noise

**DOI:** 10.3390/s22134853

**Published:** 2022-06-27

**Authors:** Bin Lin, Guoping Hu, Hao Zhou, Guimei Zheng, Yuwei Song

**Affiliations:** 1 Air and Missile Defense College, Air Force Engineering University, Xi’an 710051, China; qq1347579672@163.com (B.L.); 17792611529@126.com (H.Z.); zheng-gm@163.com (G.Z.); songyuwei2015@163.com (Y.S.); 2 Graduate College, Air Force Engineering University, Xi’an 710051, China

**Keywords:** impulse noise, low-altitude targets, multipath effects, array signal processing, direction of arrival estimation

## Abstract

Due to the discontinuity of ocean waves and mountains, there are often multipath propagation effects and obvious pulse characteristics in low-altitude detection. If the conventional direction of arrival (DOA) estimation method is directly used for direction finding, it will lead to a large error. In view of serious misalignment in the DOA estimation of multipath signals under the background of impulse noise, a DOA estimation method based on spatial difference and a modified projection subspace algorithm is proposed in this paper. Firstly, the covariance matrix of the received data vector is used for spatial difference to eliminate the multipath effects of low-altitude targets. Secondly, the modified projection matrix is constructed using the signal source estimated with the least squares criterion and then used for modifying the covariance matrix, thus eliminating the cross-covariance matrices that affect the estimation accuracy. Finally, the modified covariance matrix is used for the DOA estimation of targets. Simulations show that the proposed algorithm achieves a higher accuracy in the DOA estimation of low-altitude targets than conventional algorithms under two common impulse noise models, without requiring prior knowledge of impulse noise.

## 1. Introduction

As an important part of array signal processing, DOA estimation is a characteristic parameter commonly used in radar detection and localization. So far, a series of related studies have been carried out on DOA estimation. At the earliest, Schmidt [1] proposed the multiple signal classification (MUSIC) algorithm based on feature subspace. Later, scholars made improvements, most of which were based on the assumption of Gaussian noise. With the increasingly complex battlefield electromagnetic environment, there is more and more interference in radar detection, making it more difficult to detect targets [2,3,4]. Especially in the low-altitude environment, the diverse terrain environment will greatly weaken the energy of electromagnetic waves, always making the detection and tracking of low-altitude targets poor [5,6,7,8]. Recent studies have shown that due to the discontinuity of ocean waves and mountains, the output signal of the array exhibits obvious impulse characteristics, and the alpha-stable distribution with heavy smearing characteristics and the compound Gaussian model can effectively describe impulse noise [9,10]. Therefore, it is of great significance to develop a DOA estimation method for low-altitude targets under the background of impulse noise.

In order to solve the problem of low-altitude multipath effects, scholars have conducted in-depth research, and they have achieved many results. Li et al. [11] first proposed the use of spatial smoothing technology to deal with coherent signals in multipath effects, but in addition to aperture loss, it was only suitable for uniform arrays. In the literature [12,13,14], the spatial differencing algorithm was used for decoherence, where the accuracy was improved to some extent but the DOA estimation effect was not satisfactory under the condition of low signal-to-noise ratio (SNR). In the literature [15,16,17,18,19], a new method with a combination of time reversal technology and a generalized MUSIC was proposed for the DOA estimation of low-altitude targets, which improved the estimation accuracy of the algorithm to some extent.

Due to the absence of finite second-order moments in impulse noise, conventional higher-order cumulant algorithms cannot be used. For the purpose of direction finding under impulse noise, in the literature [20], a theory based on fractional lower-order statistics (FLOS) was first proposed. Based on this theory, Mendel et al. [21,22,23] proposed the robust covariation-based MUSIC (ROC-MUSIC) algorithm in combination with the idea of co-variation. In the literature [24,25,26], the FLOM-MUSIC algorithm based on the fractional lower-order moment (FLOM) was proposed. In the literature [27,28,29], a fractional lower-order cyclic MUSIC (FLOC-MUSIC) algorithm using a fractional lower-order cyclic covariance matrix was proposed. However, these FLOS-based algorithms require prior knowledge of the characteristic exponent of stable distribution. To this end, the infinite MUSIC (IN-MUSIC) algorithm for received data was proposed in the literature [30], which was robust for outliers, but it required large samples to obtain a satisfactory performance. In order to solve the problem of direction finding of coherent signals in the background of impulse noise, the literature [31] proposed an algorithm based on infinite norm normalization preprocessing and sparse representation. The algorithm does not need to predict the number of signal sources, and it can be well adapted to coherent sources. In the literature [32], suppression of impulse noise is achieved by using an infinite norm exponential kernel covariance matrix. In order to obtain the global optimal solution of the method, a quantum electromagnetic field optimization algorithm is designed, and the Monte Carlo experiment verifies the effectiveness of the method.

In order to improve the direction-finding accuracy of low-altitude targets under the background of impulse noise, a DOA estimation method based on a spatial difference algorithm and a modified projection subspace algorithm is proposed in this paper. This method restores the covariance matrix of the received signal to full rank through a spatial difference algorithm. The signal source vector is estimated according to the least squares criterion, whereby the modified projection matrix is constructed and the cross-covariance matrix of the signal and the noise is then calculated. The sample covariance matrix is modified to remove interference terms. Next, the best correction coefficient is obtained with the maximum likelihood criterion, followed by re-adjustment of the covariance matrix. Finally, DOA estimation is conducted using the subspace classification algorithm. Simulation results verify the effectiveness of the proposed method.

The advantages of the proposed method can be given as follows:

1. Compared with the algorithm based on fractional low-order moments, the algorithm in this paper does not require prior knowledge of the characteristic exponent of stable distribution, and it is more adaptable to the environment;
2. Compared with the algorithm in the literature [27,28,29,30,31], the algorithm in this paper does not require large sample sampling and loop iterations, so the computational complexity is low;
3. Compared with the conventional low-altitude target DOA estimation method that only deals with rank deficiencies, the proposed algorithm has better performance in low-altitude environment.

## 2. Signal Model in Low-Altitude Multipath Environment

**Figure 1:**
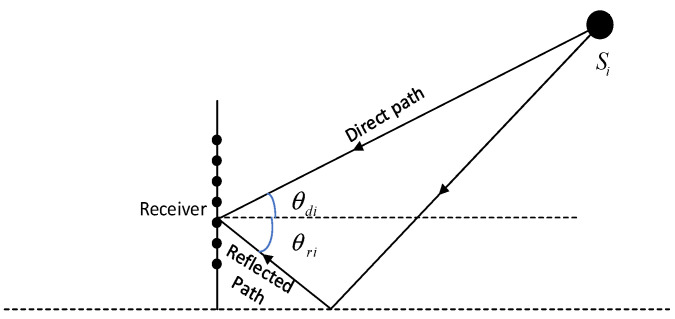
Signal reception model in low-altitude multipath environment.

The signal reception model of the array in the low-altitude multipath environment is shown in the following figure:

Suppose *K* far-field narrowband signals $s_{i}(t)(i=1,2,\ldots ,K)$ are incident on a uniform linear array with an angle of $\theta_{i}$. The spacing *d* of the array elements is one half of the signal wavelength. The array consists of *M* uniformly distributed array elements, where each element is omnidirectional. Then, the signal steering vector of the array can be expressed as:

(1) $$A=\left[a_{d1}+a_{r1},\cdots ,a_{dK}+a_{rK}\right]$$

where $a_{di}\left(\theta_{di}\right)={\left[1,e^{-j\pi \sin\theta_{di}},\cdots ,e^{-j\pi (M-1)\sin\theta_{di}}\right]}^{T}$ and $a_{ri}\left(\theta_{ri}\right)=\delta_{i}a_{di}\left(\theta_{ri}\right)$. As shown in Figure 1, $\theta_{di}$ and $\theta_{ri}$ are the incident angles after direct incidence and reflection, respectively; $\delta_{i}$ is the reflection coefficient; (·)${}^{T}$ is the transposition of the matrix.

Due to the characteristics of low-altitude multipath effects, $\theta_{di}=-\theta_{ri}=\theta_{i}$. Therefore, the signal steering vector can be expressed as:

(2) $$A=\left[a\left(\theta_{1}\right)+\delta_{1}a\left(-\theta_{1}\right),\cdots ,a\left(\theta_{K}\right)+\delta_{K}a\left(-\theta_{K}\right)\right]$$

The received signals of array can be expressed as:

(3) y(t)=As(t)+n(t)=Bx(t)+n(t)

where $s(t)={\left[s_{1}(t),s_{2}(t),\cdots ,s_{K}(t)\right]}^{T}$; $B={\left[a\left(\theta_{1}\right),a\left(-\theta_{1}\right),\cdots ,a\left(\theta_{K}\right),a\left(-\theta_{K}\right)\right]}_{N\times 2K}$; $x(t)={\left[s_{1}(t),\delta_{1}s_{1}(t),\cdots ,s_{K}(t),\delta_{K}s_{K}(t)\right]}_{2K\times 1}^{T}$; and n(t) is the impulse noise vector received by the array.

According to the above equations, it can be concluded that under ideal conditions, the covariance matrix of the received signal of array is:

(4) $$R=E\left[y(t)y^{H}(t)\right]=BR_{x}B^{H}+E\left[n(t)n^{H}(t)\right]=BR_{x}B^{H}+R_{n}=BR_{x}B^{H}+R_{n}$$

where $R_{x}$ is the covariance matrix of x(t); $R_{n}$ is the covariance matrix of noise n(t); and (·)${}^{H}$ is the conjugate transpose of the matrix.

Equation (4) can be established only when the number of snapshots approaches infinity. In actual cases, after y(t) is expanded to Bx(t)+n(t), as shown in Equation (5) where the covariance of each term is calculated, it is found that the signal–noise cross-covariance calculated matrix $R_{\mathrm{xn}}$ and the noise–signal cross-covariance matrix $R_{\mathrm{nx}}$ are non-zero matrices:

(5) $$\begin{array}{l} R=\frac{1}{N}\sum_{t=1}^{N}y\left(t\right)y^{H}\left(t\right)=\frac{1}{N}\sum_{t=1}^{N}\left(Bx\left(t\right)+n\left(t\right)\right){\left(Bx\left(t\right)+n\left(t\right)\right)}^{H}\\ =B\left[\frac{1}{N}\sum_{t=1}^{N}x\left(t\right)x^{H}\left(t\right)\right]B^{H}+\frac{1}{N}\sum_{t=1}^{N}n\left(t\right)n^{H}\left(t\right)+B\left[\frac{1}{N}\sum_{t=1}^{N}x\left(t\right)n^{H}\left(t\right)\right]+\left[\frac{1}{N}\sum_{t=1}^{N}x\left(t\right)n^{H}\left(t\right)\right]B^{H}\\ =R_{\mathrm{xx}}+R_{\mathrm{nn}}+R_{\mathrm{xn}}+R_{\mathrm{nx}} \end{array}$$

where *N* is the number of snapshots; $R_{\mathrm{xx}}$ is the signal covariance matrix; and $R_{\mathrm{nn}}$ is the noise covariance matrix.

## 3. DOA Estimation of Low-Altitude Targets under Impulse Noise

### 3.1. Decoherence with Spatial Difference Algorithm

In the low-altitude multipath environment, the direct signal and the reflected signal contained in received signals of array are correlated, as a result of which there will be angle loss in case of direct feature decomposition of R. Therefore, we use the spatial difference algorithm to divide the received signals y(t) of array into *L* sub-arrays $y_{l}(t)$ that overlap each other, and each sub-array contains *m* array elements:

(6) $$y_{l}(t)=B_{m}V^{l-1}x(t)+n_{l}(t)$$

where l=1,2,⋯,L≜M−m+1; $B_{m}$ is the first *m* rows of ***B***; and $n_{l}(t)$ represents the noise received by the *l-*th subarray. Furthermore:

(7) $$V=diag\left[v(\theta_{1}),v(-\theta_{1}),\cdots ,v(\theta_{K}),v(-\theta_{K})\right]$$

where $v(\theta_{i})=e^{-j\pi \sin\theta_{i}}$; i=1,2,⋯,K.

The covariance matrix of submatrix $y_{l}(t)$ is calculated. With Equation (5), the covariance submatrix is obtained:


(8) $$\begin{array}{l} {\hat{R}}_{l}=\frac{1}{N}\sum_{t=1}^{N}\left[y_{l}(t)y_{l}^{H}(t)\right]\\ =\frac{1}{N}\sum_{t=1}^{N}\left[[B_{m}V^{l-1}x(t)+n_{l}(t)]{\left[B_{m}V^{l-1}x(t)+n_{l}(t)\right]}^{H}\right]\\ =R_{\mathrm{xx}}^{l}+R_{\mathrm{nn}}^{l}+R_{\mathrm{xn}}^{l}+R_{\mathrm{nx}}^{l} \end{array}$$

The spatial difference matrix consists of the difference between the first covariance submatrix and the backward covariance matrix [13]:

(9) $$\hat{R}=\frac{1}{L}\underset{l=1}{\sum^{L}}\left[{\hat{R}}_{1}-J{\left({\hat{R}}_{l}\right)}^{*}J\right]$$

where J is the m×m -dimensional anti-diagonal matrix; the elements on the anti-diagonal are all 1; the rest of the elements are 0; and (·)${}^{*}$ is the conjugate of the matrix.

Since it is difficult to estimate all target angles due to the influence of low-altitude multipath effects, the spatial difference algorithm is used to eliminate this influence. However, the impulse noise component in the covariance matrix still greatly affects the estimation accuracy.

### 3.2. Modified Projective Subspace Algorithm

As the cross-covariance component in the covariance matrix $\hat{R}$ greatly reduces the accuracy of DOA estimation, we need to first estimate x(t) to remove the influence of these disturbance terms:

(10) $$\hat{x}\left(t\right)=\arg\underset{y}{\min}{\left\|y\left(t\right)-\hat{B}\left(\theta \right)x\left(t\right)\right\|}_{2}^{2}$$

where $\hat{x}\left(t\right)$ is the estimated value of x(t) and $\hat{B}\left(\theta \right)$ is the estimated value of $B\left(\theta \right)$. For the convenience of expression, $B\left(\theta \right)$ is denoted as B.

According to the least squares rule, formula (10) is minimized to obtain:

(11) $$\hat{x}\left(t\right)={\left({\hat{B}}^{H}\hat{B}\right)}^{-1}{\hat{B}}^{H}y\left(t\right)$$

Taking the difference between the observed value of the array signal and the estimated signal as an estimate of noise, we can get:

(12) $$\hat{n}\left(t\right)=y\left(t\right)-\hat{B}\hat{x}\left(t\right)$$

Then, the estimated value of the third term in Equation (8) can be obtained:

(13) $$\begin{array}{l} {\hat{R}}_{\mathrm{xn}}^{l}={\hat{B}}_{m}\left[\frac{1}{N}\sum_{t=1}^{N}\hat{x}\left(t\right){\hat{n}}^{H}\left(t\right)\right]\\ ={\hat{B}}_{m}\left[\frac{1}{N}\sum_{t=1}^{N}{\left({\hat{B}}_{m}^{H}{\hat{B}}_{m}\right)}^{-1}{\hat{B}}_{m}^{H}y\left(t\right)\left(y^{H}\left(t\right)-y^{H}\left(t\right){\hat{B}}_{m}{\left({\hat{B}}_{m}^{H}{\hat{B}}_{m}\right)}^{-1}{\hat{B}}_{m}^{H}\right)\right]\\ ={\hat{B}}_{m}{\left({\hat{B}}_{m}^{H}{\hat{B}}_{m}\right)}^{-1}{\hat{B}}_{m}^{H}\left[\frac{1}{N}\sum_{t=1}^{N}y\left(t\right)y^{H}\left(t\right)\left(I_{MN}-{\hat{B}}_{m}{\left({\hat{B}}_{m}^{H}{\hat{B}}_{m}\right)}^{-1}{\hat{B}}_{m}^{H}\right)\right] \end{array}$$

where ${\hat{B}}_{m}$ is the first *m* row of $\hat{B}$.

In order to facilitate subsequent calculation and representation, the following projection matrix is assumed:

(14) $${\hat{P}}_{B}={\hat{B}}_{m}{\left({\hat{B}}_{m}^{H}{\hat{B}}_{m}\right)}^{-1}{\hat{B}}_{m}^{H}$$

(15) $${\hat{P}}_{B}^{⊥}=I_{MN}-{\hat{P}}_{B}$$

After simplification:

(16) $${\hat{R}}_{\mathrm{xn}}^{l}={\hat{P}}_{B}\hat{R}{\hat{P}}_{B}^{⊥}$$

Similarly, we can get:

(17) $${\hat{R}}_{\mathrm{nx}}^{l}={\hat{P}}_{B}^{⊥}\hat{R}{\hat{P}}_{B}$$

(18) $${\hat{R}}_{\mathrm{nn}}^{l}={\hat{P}}_{B}^{⊥}\hat{R}{\hat{P}}_{B}^{⊥}$$

Because the steering vector matrix $\hat{B}$ is unpredictable, ${\hat{P}}_{B}$ and ${\hat{P}}_{B}^{⊥}$ cannot be calculated. Therefore, we perform eigen decomposition of the covariance matrix $\hat{R}$ after spatial differentiation:

(19) $$\hat{R}={\hat{U}}_{x}{\hat{\Sigma }}_{x}{\hat{U}}_{x}^{H}+{\hat{U}}_{n}{\hat{\Sigma }}_{n}{\hat{U}}_{n}^{H}$$

where ${\hat{\Sigma }}_{x}=diag[\lambda_{1},\ldots ,\lambda_{K}]$, ${\hat{\Sigma }}_{n}=diag[\lambda_{K+1},\ldots ,\lambda_{M}]$ and $\lambda_{1}\geq \cdots \geq \lambda_{K}>\lambda_{K}=\lambda_{K+1}=\cdots =\lambda_{M}$; ${\hat{U}}_{x}=[u_{1},u_{2},\cdots u_{K}]$, ${\hat{U}}_{n}=[u_{K+1},u_{K+2},\cdots u_{M}]$, $u_{i}$ is the eigenvector corresponding to the eigenvalue $\lambda_{i}$.

Upon calculation, it is found that the subspace generated by the signal covariance matrices ${\hat{U}}_{x}$ and $B_{m}$ is the same, that is:

(20) $$\mathrm{span}\left\{u_{1},u_{2},\cdots ,u_{K}\right\}=\mathrm{span}\left\{b_{1},b_{2},\cdots ,b_{K}\right\}$$

Hence, by assuming that there is a matrix T that makes ${\hat{U}}_{x}T=B_{m}$ established, we get:

(21) $$\begin{array}{l} {\hat{P}}_{B}={\hat{B}}_{m}{\left({\hat{B}}_{m}^{H}{\hat{B}}_{m}\right)}^{-1}{\hat{B}}_{m}^{H}\\ ={\hat{U}}_{x}T{\left[{\left({\hat{U}}_{x}T\right)}^{H}{\hat{U}}_{x}T\right]}^{-1}{\left({\hat{U}}_{x}T\right)}^{H}\\ ={\hat{U}}_{x}{\left({\hat{U}}_{x}^{H}{\hat{U}}_{x}\right)}^{-1}{\hat{U}}_{x}^{H} \end{array}$$

So, ${\hat{P}}_{B}^{⊥}$ can be represented as:

(22) $${\hat{P}}_{B}^{⊥}=I_{MN}-{\hat{P}}_{B}$$

Formulas (21) and (22) are substituted into Formulas (17) and (18) to get:

(23) $${\hat{R}}_{\mathrm{xn}}^{l}={\hat{P}}_{B}^{⊥}\hat{R}{\hat{P}}_{B}$$

(24) $${\hat{R}}_{\mathrm{nx}}^{l}={\hat{P}}_{B}^{⊥}\hat{R}{\hat{P}}_{B}^{⊥}$$

${\hat{R}}_{l}$ and $\hat{R}$ after removal of disturbance terms can be expressed as:

(25) $${\hat{R}}_{l}^{\prime }=\left[{\hat{R}}_{l}-\varepsilon \left({\hat{R}}_{xn}^{l}+{\hat{R}}_{nx}^{l}\right)\right]{\left[{\hat{R}}_{l}-\varepsilon \left({\hat{R}}_{xn}^{l}+{\hat{R}}_{nx}^{l}\right)\right]}^{H}$$

(26) $${\hat{R}}^{\prime }=\frac{1}{L}\underset{l=1}{\sum^{L}}\left[{\hat{R}}_{1}^{\prime }-J{\left({\hat{R}}_{l}^{\prime }\right)}^{*}J\right]$$

in Equation (25), $\varepsilon \in \left[0,1\right]$ is used to correct the estimation error. If ${\hat{R}}_{xn}^{l}$, ${\hat{R}}_{nx}^{l}$ are equal to $R_{xn}^{l}$, $R_{nx}^{l}$, respectively, ε=1. In practice, since the error of simulation estimation cannot be accurately predicted, a specific uniform step size is substituted into the following equation for calculation:

(27) $$f(\varepsilon )=\underset{\varepsilon }{\min}\ln\det({\hat{P}}_{B}{}^{\prime }\hat{R}{\hat{P}}_{B}{}^{\prime }+\frac{\mathrm{Tr}\{{\hat{P}}_{B}^{{⊥}^{\prime }}\hat{R}\}}{m-K}{\hat{P}}_{B}^{{⊥}^{\prime }})$$

according to the maximum likelihood criterion, ε is the best correction coefficient when f(ε) reaches the minimum value. In this equation, ${\hat{P}}_{B}{}^{\prime }$ is the modified projection matrix of ${\hat{R}}^{\prime }$ obtained by eigenvalue decomposition; ${\hat{P}}_{B}^{⊥}{}^{\prime }=I_{MN}-{\hat{P}}_{B}{}^{\prime }$ and $\mathrm{Tr}\left\{{\hat{P}}_{B}^{⊥}{}^{\prime }\hat{R}\right\}$ is the trace of the matrix.

Finally, the DOA of the target is obtained through eigenvalue decomposition of ${\hat{R}}^{\prime }$ using the MUSIC algorithm.

## 4. Basic Steps of the Algorithm and Complexity Analysis

### 4.1. The Basic Steps of the Algorithm

According to the above analysis, the algorithm in this paper is summarized as follows:

1. Calculate the data covariance matrix R of the array element output vector y(t);
2. As shown in Equations (6)–(9), spatial difference operation is performed to obtain $\hat{R}$;
3. Eigenvalue decomposition of $\hat{R}$ is performed to get ${\hat{U}}_{x}$;
4. Calculate the modified projection matrix ${\hat{P}}_{B}$ and ${\hat{P}}_{B}^{⊥}$ with Equations (21) and (22);
5. As shown in Equations (23)–(26), the cross-covariance matrices of ${\hat{R}}_{\mathrm{xn}}^{l}$ and ${\hat{R}}_{\mathrm{nx}}^{l}$ are constructed to correct the estimated value of ${\hat{R}}_{l}$;
6. As shown in Equation (27), the optimal correction coefficient ε is obtained using the maximum likelihood criterion, and the estimated value of ${\hat{R}}_{l}$ is re-adjusted;
7. Finally, DOA estimation is performed for the adjusted ${\hat{R}}_{l}^{\prime }$ using MUSIC algorithm.

### 4.2. Algorithm Complexity Analysis

Let’s assume that the number of array elements is *M*; the number of signal sources is *K*; the number of sampling points is *N*; the number of angle searches is $N_{\theta }$, and *E* is the number of ε divided by a uniform step size. The computational complexity of steps 1~5 of this algorithm is approximately $\left(E+2N+1\right){\left(M\right)}^{3}+\left(EN+K\right){\left(M\right)}^{2}+K^{2}M+K^{3}$. The complexity of spatial spectrum calculation and search in steps 6~7 is approximately $O\left(\left(M-K\right)MN_{\theta }\left(E+1\right)\right)$. Therefore, the total complexity of the algorithm herein is approximately $O\left(\begin{array}{l} \left(E+2N+1\right){\left(M\right)}^{3}+\left(EN+EN_{\theta }+N_{\theta }+K\right){\left(M\right)}^{2}\\ +\left(K^{2}-KEN_{\theta }-KN_{\theta }\right)M+K^{3} \end{array}\right)$.

## 5. Simulation Results and Analysis

### 5.1. Impulse Noise Model

Compared with Gaussian noise, the probability distribution of impulse noise is characterized by long tailing. Typical impulse noise models include a Gaussian mixture model (GMM), noise model, and SαS noise model.

The GMM noise model used in the simulation consists of two Gaussian components, and its probability density function (PDF) is:

(28) $$p_{n}(x)=\underset{i=1}{\sum^{2}}\frac{c_{i}}{\pi \sigma_{i}^{2}}\exp\left(-\frac{|x{|}^{2}}{\sigma_{i}^{2}}\right)$$

where i=1,2, $0\leq c_{i}\leq 1$ is the probability of the *i*-th term and $c_{1}+c_{2}=1$; $\sigma_{i}^{2}$ is the variance of the *i-*th term; it is set in the simulation that $\sigma_{2}^{2}=100\sigma_{1}^{2}$. Meanwhile, the signal-to-noise ratio (SNR) is defined as:

(29) $$SNR=\frac{\sigma_{s}^{2}}{\sigma_{1}^{2}}$$

the noise power should have been $c_{1}\sigma_{1}^{2}+c_{2}\sigma_{2}^{2}$, but since $\sigma_{1}^{2}$ is set to a large noise background in this simulation, the presence of $\sigma_{2}^{2}$ is considered an outlier.

As the most commonly used impulse noise distribution model, the SαS noise model PDF cannot usually be given in closed form, but its characteristic function is:

(30) $$\phi (x)=\exp\left(-\gamma^{\alpha }|x{|}^{\alpha }\right)$$

where α is the characteristic exponent and 0<α≤2. The larger the α, the weaker the pulse characteristic. When α=2, it degenerates into a Gaussian distribution. The dispersion coefficient is γ, and it is similar to the variance in a Gaussian distribution. Since the common SNR is nonsense for the SαS distribution with α<2, the generalized SNR (GSNR) is used:

(31) $$GSNR=\frac{\sigma_{s}^{2}}{\gamma^{\alpha }}$$

A uniform linear array with 35 array elements is used in the simulation. Each subarray contains 30 array elements, that is, *L* = 6. The angles of two incoherent narrowband signals are 2° and 6°. The signal wavelength $\lambda =\frac{d}{2}$; the array element spacing *d* = 2; the source power is equal to $\sigma_{s}^{2}$; the reflection coefficient $\delta_{i}$ = 0.9; and the number of snapshots is 200.

### 5.2. Spatial Spectrum Estimation

**Figure 2:**
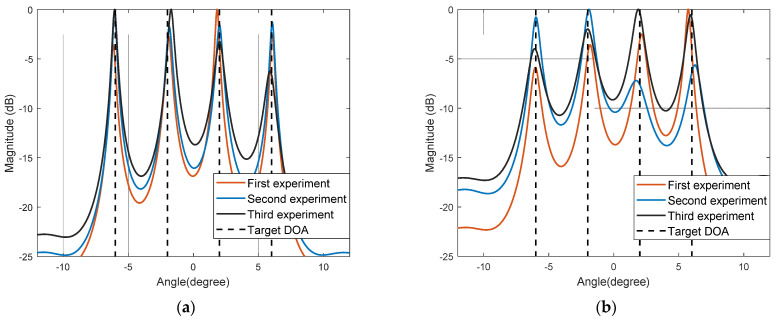
Algorithm space spectrum under the background of GMM noise: (**a**) $c_{2}$ = 0.1; (**b**) $c_{2}$ = 0.3.

Experiment one shows the spatial spectrum estimation effect with the simulation algorithm under the GMM noise model. Figure 2 shows the spatial spectrum of three independent experiments with the algorithm, when the probability of outliers is $c_{2}$ = 0.1, $c_{2}$ = 0.3, and SNR = 5 dB.

Figure 2 shows that under the background of GMM noise, when the probability of outliers is 0.1, the target direction can be accurately estimated with the algorithm. When the probability of outliers rises to 0.3, although specific spectral peaks are not steep, the algorithm still maintains a high resolution.

Experiment two shows the spatial spectrum estimation effect with the simulation algorithm under the SαS noise model. Figure 2 shows the spatial spectrum of three independent experiments with the algorithm, when the characteristic exponent α=1.6 and α=1.3, where GSNR = 5 dB.

**Figure 3:**
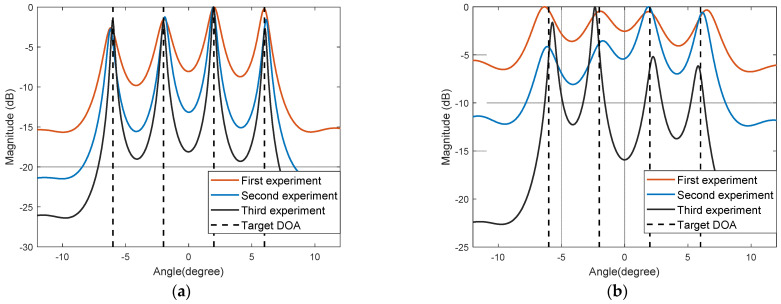
Spatial spectrum under noise background with the algorithm: (**a**) α=1.6; (**b**) α=1.3.

Figure 3 shows that under the background of SαS noise, when the characteristic exponent α decreases, the impact characteristic of the noise is enhanced and the position of the spectral peak deviates from the target direction to a certain extent. It can be learned from the spectral peak search that the algorithm can still maintain a high accuracy and a strong robustness.

### 5.3. Comparative Analysis of DOA Estimation Performance

In this section, simulation experiments are carried out to study the performance of this algorithm. Since the MUSIC algorithm fails to effectively identify coherent signal sources, the spatial smoothing method [11] is used in the following comparison algorithms. The comparison algorithms being compared include ROC-SSMUSIC [22], FLOM-SSMUSIC [24], FLOC-SSMUSIC [27], and IN-SSMUSIC [31].

The root mean square error (RMSE) of DOA estimation is defined as:

(32) $$\mathrm{RMSE}=\sqrt{1/2N_{mont}\underset{i=1}{\sum^{N_{mont}}}\underset{k=1}{\sum^{K}}\left({\left|\theta_{i}-\theta_{ik}\right|}^{2}\right)}$$

where $N_{mont}$ is the number of Monte Carlo experiments; *K* is the number of signals; and $\theta_{i}$ and $\theta_{ik}$ are the actual value and the estimated value of the *i*-th signal azimuth in each experiment.

A successful estimation of each angle is defined as:

(33) $$\left|\theta_{i}-\theta_{ik}\right|\leq 2{}^{\circ},k=1,2,\cdots K$$

namely, the difference between the estimated value of the angle and the actual value is less than 2°. The probability of success is the ratio of the number of successes to the total number of estimations.

**Figure 4:**
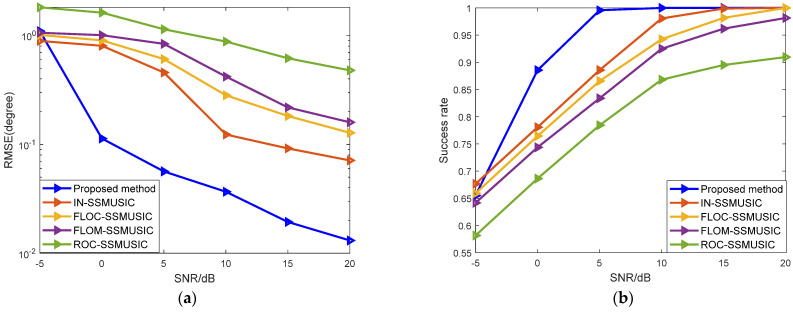
Analysis of mean square error and success rate under GMM noise: (**a**) Relationship between RMSE and SNR; (**b**) Relationship between success rate and SNR.

In experiment three, the number of Monte Carlo experiments was 500, and other settings were the same as above. Different SNRs were set to compare the estimation performance under the background of GMM noise. Figure 4a shows the curve of the changes of RMSE with five different algorithms, when the SNR changes from −5 dB to 20 dB at a step of 5 dB. Figure 4b shows the curves of the success rate of the five algorithms with changes of SNR.

As can be seen from Figure 4, the theoretical method of edge asymptotic fitting of the covariant matrix adopted in the ROC-SSMUSIC algorithm is not very effective. Due to the difference between the impulse noise model and the selection of noise parameters, the estimation performance of the FLOM-SSMUSIC algorithm and the FLOC-SSMUSIC algorithm is significantly improved when the SNR is greater than −5 dB. With pre-treatment of infinite norm normalization to reduce the influence of impulse noise, the success rate of the IN-SSMUSIC algorithm is closest to the algorithm in this paper when the SNR is high. The proposed algorithm basically achieves the best estimation performance within the entire SNR simulation range, and especially when the SNR is greater than 0 dB, the estimation performance herein is significantly better than other algorithms.

**Figure 5:**
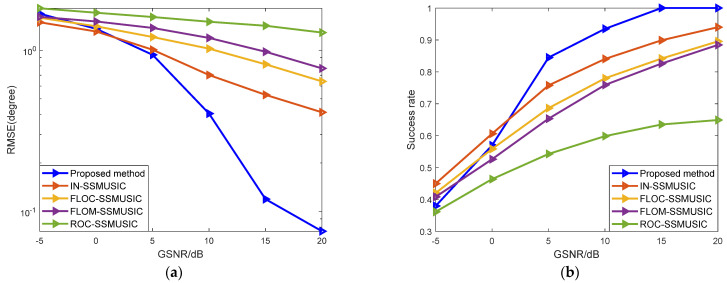
Analysis of the mean square error and success rate of the algorithm under noise: (**a**) Relationship between RMSE and GSNR; (**b**) Relationship between success rate and GSNR.

In experiment four, while the simulation settings in this section remain unchanged, different GSNRs were set under the background of SαS noise to compare the algorithm’s performance, where α = 1.4. Figure 5a shows the curve of changes of RMSE with the five different algorithms when the GSNR changes from −5 dB to 20 dB at a step of 5 dB. Figure 5b shows the curves of the success rate of the five algorithms with changes of GSNR.

It can be seen from Figure 5 that with increased GSNR, the impact of impulse noise decreases and the success rate of DOA estimation is significantly improved. Low-order fractional moment algorithms such as FLOM-SSMUSIC and FLOC-SSMUSIC achieve better performance at low GSNR, but such algorithms depend too much on prior knowledge of noise. The performance of the proposed algorithm is better than the other algorithms being compared. However, in the case of a low GSNR, due to the strong impulse noise impact, its estimation accuracy decreases to a certain extent.

**Figure 6:**
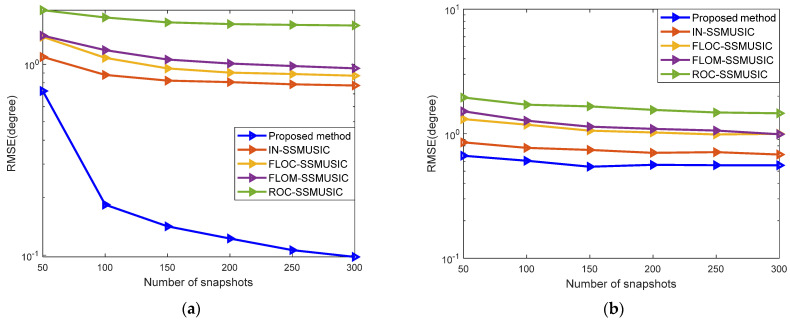
Influence of the number of snapshots on the performance of the algorithm: (**a**) GMM noise; (**b**) SαS noise.

In Experiment five, the influence of the number of snapshots on the performance of the algorithm was analyzed. The performance of the algorithm was compared by setting a different number of snapshots under two different noise models. Figure 6a shows the curve of changes of RMSE with the five different algorithms when the number of snapshots changes from 50 to 300 at a step of 50 under the background of GMM noise with SNR = 5 dB. Figure 6b shows the change of RMSE with the five algorithms when the number of snapshots changes from 50 to 300 at a step of 50 under the background of SαS noise with GSNR = 5 dB and α=1.4.

It can be seen from Figure 6 that with increased snapshots, the estimation performance of each algorithm is enhanced to a certain extent either under a GMM or an SαS noise model, and it gradually becomes stable. Regardless of the number of snapshots, the RMSE of the proposed algorithm is lower than other algorithms. Under the GMM noise, however, when the number of snapshots is less than 100, the performance of the proposed algorithm is poor. This is because when the number of snapshots is small, it is difficult for the sample-based estimation algorithm to correctly separate the signal and the noise subspaces, resulting in a larger error.

**Figure 7:**
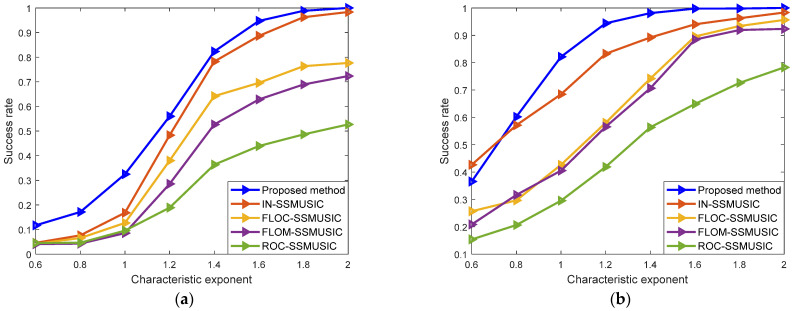
Influence of characteristic exponent on algorithm performance: (**a**) GSNR = 10 dB; (**b**) GSNR = 20 dB.

In Experiment six, the influence of the characteristic exponent α on the performance of the algorithm under the SαS noise background was analyzed. While the simulation in this section remains unchanged, different characteristic exponents are set. Figure 7 shows the curves of the success rate of five different algorithms when the characteristic exponent changes from 0.6 to 2 at a step of 0.2 when GSNR = 10 dB and GSNR = 20 dB.

Since the larger the characteristic exponent, the weaker the impulsiveness of the noise, the success rate of these algorithms is improved with increased characteristic exponent. It can be seen from the figure that the performance of ROC-SSMUSIC algorithm is the worst in an SαS noise environment, without a significant effect on the change of the characteristic exponent. The FLOM-SSMUSIC, FLOC-SSMUSIC, and IN-SSMUSIC algorithms are sensitive to the change of the impulse noise intensity to some extent, that is, the reduction of the noise intensity reduces RMSE. When the characteristic exponent of impulse noise contained in the output signal of the array changes from 1.6 to 2, the above three algorithms are insensitive to the change of impulse noise intensity, and they show strong robustness. However, the algorithm herein maintains a strong estimation performance in a 10 dB or a 20 dB SαS noise environment.

## 6. Conclusions

As to direction finding of low-altitude targets under the background of impulse noise, a DOA estimation method based on spatial difference and on a modified projection subspace algorithm is proposed. In this method, the covariance matrix is first constructed with the received signal vector, and then the spatial difference operation is performed to eliminate the influence of low-altitude multipath. Next, the array signal vector is estimated according to the least squares criterion, after which the modified projection matrix is constructed to calculate the cross-covariance matrix of the signal noise. Then, the covariance matrix of the samples is modified to remove the disturbance terms, thus completing the DOA estimation of the targets. The experimental results show that the proposed algorithm achieves satisfactory DOA estimation of low-altitude targets under different impulse noise backgrounds, with higher accuracy and more practical application compared with conventional algorithms. In the next step, on the basis of guaranteeing the performance of the algorithm, the extended study of the spatial difference method for coherent and incoherent mixed signals in the background of impulse noise will be carefully considered.

## Data Availability

The data presented in this study are available on request from the corresponding author. The data are not publicly available due to being obtained from the simulation of the signal models listed in the article and not from publicly available datasets.
